# Modelling and Control Design of a Non-Collaborative UAV Wireless Charging System

**DOI:** 10.3390/s22207897

**Published:** 2022-10-17

**Authors:** Oussama Allama, Mohamed Hadi Habaebi, Sheroz Khan, Elfatih A. A. Elsheikh, F. M. Suliman

**Affiliations:** 1 IoT and Wireless Communication Protocols Laboratory, Department of Electrical and Computer Engineering, International Islamic University Malaysia (IIUM), Kuala Lumpur 53100, Malaysia; 2 Department of Electrical and Renewable Energy Engineering, Unaizah Colleges of Engineering, Unaizah 56453, Saudi Arabia; 3 Department of Electrical Engineering, College of Engineering, King Khalid University, Abha 61421, Saudi Arabia

**Keywords:** 3D omnidirectional, WPT maximization, extremum seeking control, magnetic tracking

## Abstract

This study proposes an analytical model of a WPT system with three orthogonal transmitter coils organised to produce a concentrated and controlled omnidirectional magnetic field suited for charging a moving, rotating load, providing maximal energy transfer without receiving end feedback. In order to create a realistic 3D WPT simulation system and a precise controller design, the mutual coupling values in terms of the receiver angular positions are modelled using the Ansys software. In using the established model of the 3DWPT system, an extremum seeking control (ESC) is used to maximize the power transfer utilizing the input power as an objective function assigned with specified parametric values defining the WPT model. The output power transmitted by the sending-end coils to a load of a moving UAV rotating in orbit is displayed. According to simulation results, when the receiver UAV speed is close to 2250 deg/s, the controller can accomplish a maximum power transfer of 2.6w in almost 1ms.

## 1. Introduction

WPT is an attractive battery-less solution for the Internet of Things (IoT), where the network of devices collects to share data wirelessly. WPT reduces the size of sensors and electrical devices by eliminating the physical connection between the transmitters and the receiver. It allows charging a wide variety of devices in both industrial and quotidian realms [1]. It is a game-changing technology for automating the recharging process for unmanned aerial vehicles (UAVs) or drones, particularly those employed in agriculture [2], crowd management [3], traffic monitoring in big cities [4], and disaster management [5]. The major limitation of WPT technology based on inductive coupling is the inability to maintain a high rate of wireless power transmission efficiency. In order to overcome these challenges, numerous coil-based topologies have been investigated.

A three orthogonal coils topology was proposed to pick up wireless power in robot applications. The primary and secondary coils were designed to fit inside a robot elbow to achieve a maximum coupling coefficient when the robot arm rotates [6]. A multi-coil structure WPT energy harvester was developed to alleviate the misalignment issue for mobile devices [7]. A cubic magnetic dipole coils arrangement was also proposed in [8] for omnidirectional and multi-load power transmission. Another study showed a 3D wireless charging cylinder with two orthogonal coils that can generate a spinning magnetic field to charge numerous loads [9]. The system of multiple bowl-shaped transmitting coils reported by the authors in [10] can create a uniform magnetic field inside the charging region to compensate for the misalignment issue. The authors developed a re-designed terminal receiver structure in [11] to improve the efficiency of an omnidirectional WPT system using three orthogonal coil structures. According to the experimental results, the quadrature-shaped pickup receiving coil structure can increase load power by up to 240%. In [12], a source coil is linked to many receivers deployed randomly in the magnetic field range of the transmitter using a three-orthogonal coil topology and an inductive coupling approach.

However, increasing the rotating magnetic field to cover many loads in space can lower WPT efficiency due to magnetic field leakage concerns [8,12]. To address the challenges mentioned above and help WPT development, researchers proposed the idea of beaming the magnetic field to the specified location. Compared to the rotational WPT, this new approach permits directional control of the generated magnetic field by adopting a particular topology at the transmission side, reducing magnetic flux leakage and enhancing efficiency [13]. The omnidirectional power transfer concept has been developed to maximise power delivery. The target load is magnetically tracked in [14], utilising an energy-beaming approach at the transmission end. By regulating the current amplitude in the crossed antenna structures at the transmitting end, the authors offered the potential of directing the magnetic field towards a specific position. In [15], the control direction of the generated magnetic field based on three orthogonal coil transmitter topologies is achieved by two proposed control techniques: the mathematical method and the two-dimensional plane method. However, both control approaches neglect receiver navigation and do not employ closed-loop time response. The same concept of the directional magnetic field is used with different control approaches [16].

Nonetheless, this research concentrated on static loads, and it requires a considerable amount of data to scan, evaluate, and compare the data of each point surrounding the sphere, which consumes a lot of resources and makes it unsuitable for intelligent system applications. Other control approaches are employed to obtain higher efficiency [17]. Still, the research does not include load mobility, and the receiver must be calibrated in three separate modes before the power control procedure can begin. Controlled omnidirectional WPT for a moving target was studied in [18], using gradient descent as a control technique. However, the paper does not discuss the speed and trajectory of movement. Furthermore, the research covers only the 2DWPT scenario.

In this work, we introduce a comprehensive analytical modelling and control design for a three-dimensional WPT system that is intended to charge a moving receiving load spinning on an orbital trajectory continuously or frequently in the three-dimensional plan. Ansys software is used to simulate the dynamic system and compute the rotating mutual coupling function of the 3D system. The results are consistent with those of the benchmark studies.

The derived formula of the angular mutual coupling enables a precise control design and tuning as well as the system closed-loop response analysis. The founded model can simulate the receiver’s continuous trajectory movement, velocity, and acceleration versus the input power variation.

The novelty of this paper lies in the development of a comprehensive analytical model for the 3DWPT system. Unlike the previous studies, our model can produce an accurate relationship between the angular position of the drone and the input power of the system. Further, the model can simulate any desired trajectory for the moving receiver around the transmitter. Unlike the previous studies, this model can simulate static and changing movement positions of the receiver.

The unique produced results of the 3D input power in the Cartesian plane, as well as the load current and the overall trajectory maximum input power, prove the novelty of the model. Further, the model was benchmarked with the previous experimental works. Additionally, having a comprehensive model of the system that can simulate a continuous movement of the drone provided an accurate control design to maximize the transferred power for a real movement of the drone. This makes the proposed model unlike the previous research studies where the controller was unrealistically designed based on two positions of the receiver.

The contribution of this paper is also presented in the implementation of the extremum seeking control technique for a 3DWPT system with circular coils, where the multi-parameter controller can maximise the input power of the system by controlling two parameters at the same time (*θ*, *β*) with an unknown reference function of the moving receiver. Eventually, the proposed control ESC technique can maximise the power transfer regardless of the position variation and velocity of the load.

Eventually, the proposed control ESC technique can maximise the power transfer regardless of the position variation and velocity of the load. The novel developed model considers the receiver’s continuous rotational movement at a variable speed by expressing the input power of the 3DWPT system in terms of the receiver position angles.

Furthermore, the extremum seeking control technique was employed to maximise the power transfer when the receiver rotates at high velocity. The simulation results of this work are validated with those experimental results in [16]. The proposed model can simulate an average angular speed of 2250 deg/s, and the designed controller can provide maximum power delivery with a response time of less than 1ms. The remaining parts of the paper are structured as follows: Section 2 contains an analysis of the parameters of the three-dimensional WPT system, including the derivation of the relationship between the input power and the location of the mobile receiver angles. Section 3 explains extremum seeking control (ES) algorithms and the mobile receiver tracking process as the result of Section 2. Section 4 concludes the paper with a quantified conclusion of this research.

## 2. Mathematical Analysis and Modelling of 3D WPT System

### 2.1. System Modelling

**Figure 1:**
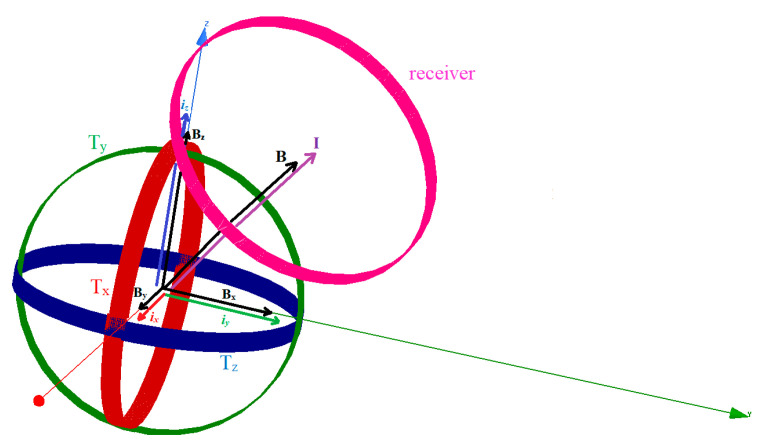
The 3D WPT system magnetic and electric resultant vectors.

The 3DWPT system architecture is based on an orthogonal topology with three identical circular sending coils T_x_, T_y_, and T_z_, as shown in Figure 1. By using an orthogonal structure for the transmitter side, the mutual coupling between the three sending coils can be cancelled.

We assume that the receiving coil is rotating in a spherical surface trajectory 𝝘 around the transmitter with a fixed radius (r = 0.3 m). In order to employ the resonant inductive WPT mechanism, a serial capacitor and resistor are added for each transmitting coil circuit.

The magnetic fields B_x_, B_y_, and B_z_ are produced, correspondingly, by the currents flowing in each transmitting coil, which are defined as *i_x_*, *i_y_*, and *i_z_*. The three transmitting end coils’ resulting current vector *I* and magnetic field vector B are related, as deduced in [19].

(1) $$B=\frac{\mu_{0}I}{4\pi r^{2}}\oint dl\cdot \left(\hat{i}\times \hat{r}\right)=\frac{\mu_{0}I}{2r}\cdot \left(\hat{i}\times \hat{r}\right)$$

Using Biot–Savart law, we can express the generated summation of the three orthogonal coil’s magnetic fields using Equation (2):

(2) $$B=B_{x}+B_{y}+B_{z}=\frac{\mu_{0}}{2r}\cdot \left(i_{x}\hat{i}+i_{y}\hat{j}+i_{z}\hat{k}\right)$$

**Figure 2:**
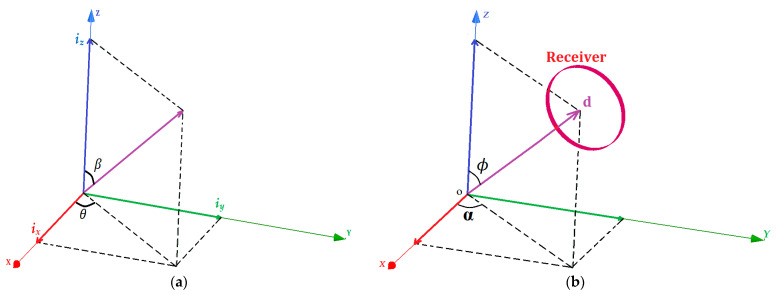
(**a**) The representation of the AC currents flowing in the 3D transmitting coils in the spherical coordinates. (**b**) The representation of the receiver position in the spherical coordinates.

We can deduce from Equation (2) that the components of the resultant vectors of the magnetic field, B, and the current, *I*, have the same direction as the unity vectors of the Cartesian plane. As a result, the magnetic field can be directed without the transmitter moving. By tuning the current amplitudes of the sending end, we can enable the generation of a directed magnetic field towards any desired point in the Cartesian plane. As illustrated in Figure 2a, we define *θ* as the magnetic resultant polar angle and *β* as the azimuthal magnetic angle in spherical coordinates. As shown in Figure 2b, α and *ϕ* are the polar and the azimuthal angles, respectively, of the receiver’s position in the spherical coordinates as well.

The three transmitting coils (T_x_, T_y_, and T_z_) of the WPT system produce the resultant magnetic and electric vectors B and *I* represented by the angular direction *θ* and *β* in the spherical coordinates. The relationship between the electric resultant vector *I* and the currents *i_x_*, *i_y_*, and *i_z_* flowing in the transmitting coils (T_x_, T_y_, and T_z_), respectively, is described by Equation (3).

(3) $$\left\{ \begin{array}{l} \begin{array}{c} i_{x}=I\times \sin\beta \cos\theta \\ i_{y}=I\times \sin\beta \sin\theta \end{array}\\ i_{z}=I\times \cos\beta \end{array}\right.$$

**Figure 3:**
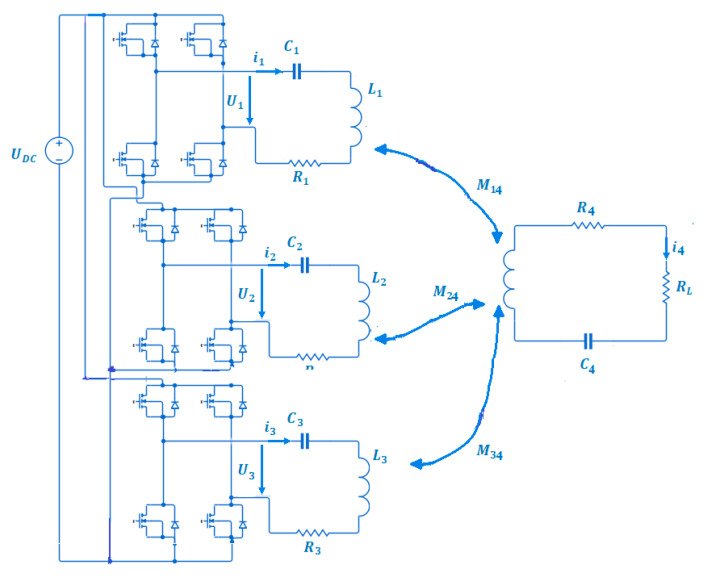
The equivalent circuit modelling of the 3D WPT system.

Here, we realise that controlling *θ* and *β* allows the targeting of any moving load in 3D space by pointing the resulting magnetic and electric field vectors at any point in the (x, y, z) plane. The electric circuit diagram of the 3D WPT system in Figure 3 is used to derive the electric mathematical model of the system.

The electric currents flowing in the sending coils T_x_, T_y_, and T_z_ are replaced by *i*_1_, *i*_2_, and *i*_3_, respectively, throughout the entire rest of the article. The system’s electric parameters are related mathematically using Equation (4).

(4) $$\{\begin{array}{c} i_{1}\left(R_{1}+jwL_{1}+\frac{1}{jwC_{1}}\right)-jwM_{14}i_{4}=U_{1}\\ \begin{array}{l} i_{2}\left(R_{2}+jwL_{2}+\frac{1}{jwC_{2}}\right)-jwM_{24}i_{4}=U_{2}\\ i_{3}\left(R_{3}+jwL_{3}+\frac{1}{jwC_{3}}\right)-jwM_{34}i_{4}=U_{3} \end{array}\\ i_{4}\left(R_{4}+R_{L}+jwL_{4}+\frac{1}{jwC_{4}}\right)-jwM_{14}i_{1}-jwM_{24}i_{2}-jwM_{34}i_{3}=0 \end{array}$$

The inverter blocks in Figure 3 generate alternating voltages U_1_, U_2_, and U_3_, which cause the transmitting coils (T_x_, T_y_, and T_z_) to exhibit the formation of the AC currents *i*_1_, *i*_2_, and *i*_3_, respectively. At the same time, the receiving load experiences an induced current i4. The three parameters, M_13_, M_23_, and M_33_, related to the coils T_x_, T_y_, and T_z_ concerning the receiver, are used to quantify the mutual coupling between the transmitting end and the receiving end. The self-inductances (L_1_, L_2_, L_3_), resistors (R_1_, R_2_, R_3_), and capacitances (C_1_, C_2_, C_3_) of the three transmitting loops are set to be equal for mathematical simplicity reasons. By changing (3) into (4) to become (5), we can introduce the angular direction parameters Theta and Betha as well as the resulting electrical vector *I* in one set of system equations, allowing us to manipulate the magnetic field resultant vector using the electric currents.

(5) $$\{\begin{array}{l} I\sin\beta \cos\theta \left(R_{1}+jwL_{1}+\frac{1}{jwC_{1}}\right)-jwM_{14}i_{4}=U_{1}\\ \begin{array}{l} I\sin\beta \sin\theta \left(R_{2}+jwL_{2}+\frac{1}{jwC_{2}}\right)-jwM_{24}i_{4}=U_{2}\\ I\cos\beta \left(R_{3}+jwL_{3}+\frac{1}{jwC_{3}}\right)-jwM_{34}i_{4}=U_{3} \end{array}\\ \begin{array}{l} i_{4}\left(R_{4}+R_{L}+jwL_{4}+\frac{1}{jwC_{4}}\right)-jwM_{14}I\sin\beta \cos\theta \\ -jwM_{24}I\sin\beta \sin\theta -jwM_{34}I\cos\beta =0 \end{array} \end{array}$$

From (5), we can deduce the final expression of the load current $i_{4}$ given in (6).

(6) $$\begin{array}{l} i_{4}=\frac{jwM_{14}I\sin\beta \cos\theta +jwM_{24}I\sin\beta \sin\theta +jwM_{34}I\cos\beta \;}{R_{4}+R_{L}+jX_{4}}\\ =\frac{jwI(M_{14}\sin\beta \cos\theta +M_{24}\sin\beta \sin\theta +M_{34}\cos\beta )\;}{R_{4}+R_{L}+jX_{4}} \end{array}$$

where $X_{4}=\left(wL_{4}-\frac{1}{jwC_{4}}\right)$.

**Figure 4:**
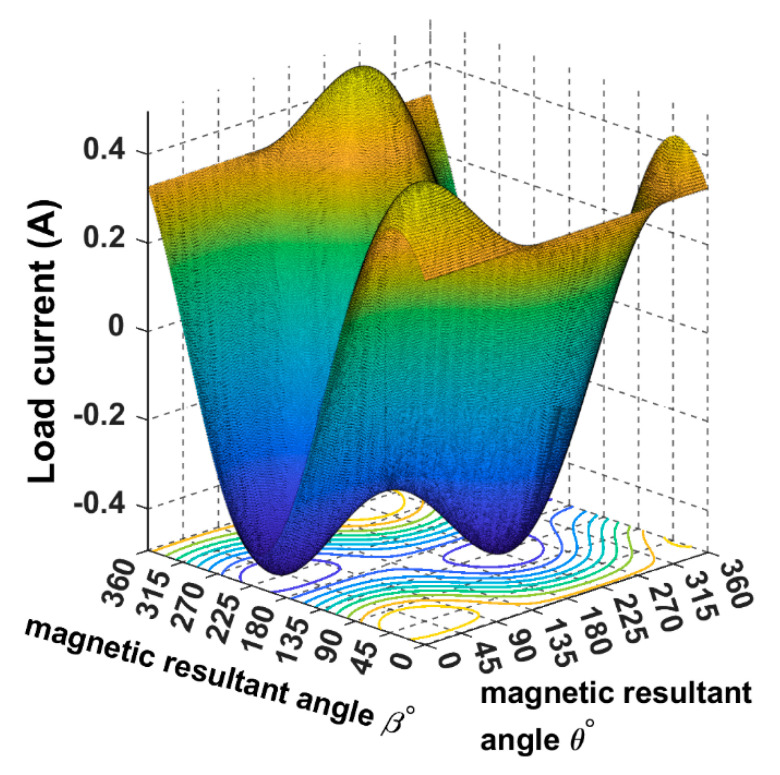
The load current when the magnetic field rotates around the origin and the receiver is placed at α = *ϕ* = 45°.

As we can notice, the load current reaches its positive maximum when both the rotating magnetic field angles *θ*° and *β*° are directed to 45°, as shown by the yellow contour in Figure 4. When the magnetic field angles are directed to the opposite position of the receiver (225°), the direction of the load current will be reversed—resulting in a negative pick as it is showing the blue contour.

**Figure 5:**
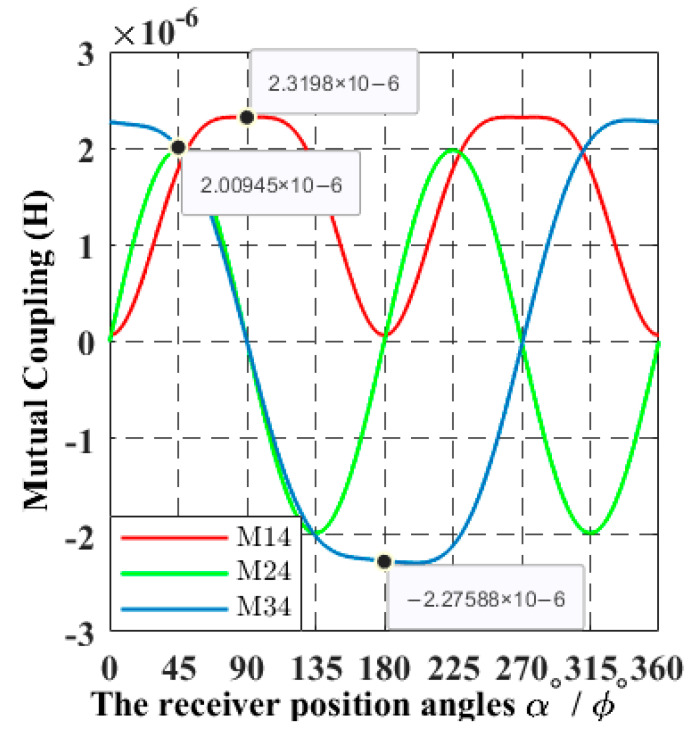
The mutual coupling curves when the receiver coil position angles α and *ϕ* are rotating from 0° to 360° through a 1° step change at a distance of 0.3 m.

**Figure 6:**
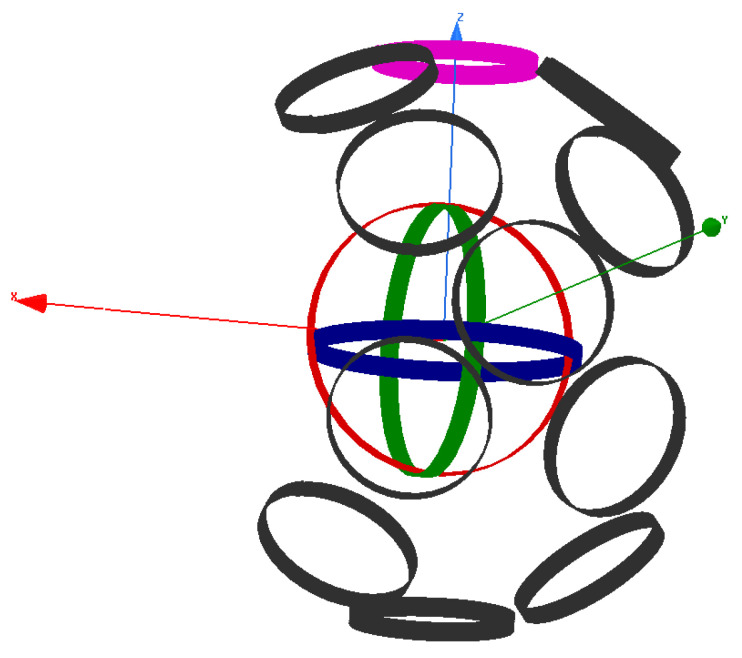
3D WPT Ansys software simulation when the receiver is rotating in a range of 0° to 360°.

Since the motion of the receiver is in a continuous trajectory, it is necessary to formulate the mathematical relationship between the continuous mutual coupling functions of the system and the spherical coordinates functions of the receiver position angles α(t) and *ϕ*(t). The three mutual coupling receiver trajectory functions in relation to the sending coils *M*_14_(α, *ϕ*), *M*_24_(α, *ϕ*), and *M*_34_(α, *ϕ*) are obtained using Ansys software finite elements calculations, as shown in Figure 5. Figure 6 shows a few positions of the receiver from the overall simulated trajectory.

### 2.2. Model Benchmarking

As we can notice from Figure 6, the starting point of the receiver movement is represented in pink colour with the spherical coordinates of (r = 0.3m, α = 0°, *ϕ* = 0°). By fixing r and changing α and *ϕ* with a 1° step from 0 to 360°, we have 360 calculated values for each mutual coupling parameter (the black coils represent the positions occupied by the receiver when moving in 𝝘 trajectory), which is sufficient to fit accurately in a continuous function *M* (α, *ϕ*). Figure 6 represents the obtained mutual coupling values in terms of the receiver angular positions α and *ϕ*. To measure the performance of the proposed model and benchmark the results, the system parameters used for the Ansys software simulation are the ones in [16], as shown in Table 1.

In Table 2, the mutual coupling values between the T_z_ coil and the receiver M_z_-L_2_ imply that Ref. [16] considered the receiver to be only present in the (x, y) plane. In contrast, in Table 3, the receiver’s movement occurs in a 3D trajectory.

As we can see, both results are in close agreement, and the small existing inequality is due to the difference in the orbit and the planes. In the first case, when *θ* = 45°, the receiver is coupled equally only with Tx and Ty in the 2D plane [16]. In this work, the receiver is simultaneously coupled equally with the three coils for the same position. The remaining two cases of the two tables are when the receiver is coupled with one coil at a time.

By applying the Fourier transform, it is possible to approximate the equations of the mutual coupling curves in Figure 5 by the functions (7)–(9) as shown:

(7) $$\begin{array}{r} M_{14}(\alpha ,\phi )=a_{01}+a_{11}\cos(\alpha ,\phi )\omega +b_{11}\sin(\alpha ,\phi )\omega +a_{21}\cos2(\alpha ,\phi )\omega \\ +b_{21}\sin2(\alpha ,\phi )\omega +a_{31}\cos3(\alpha ,\phi )\omega +b_{31}\sin3(\alpha ,\phi )\omega \end{array}$$

(8) $$\begin{array}{r} M_{24}(\alpha ,\phi )=a_{02}+a_{12}\cos(\alpha ,\phi )\omega +b_{12}\sin(\alpha ,\phi )\omega +a_{22}\cos2(\alpha ,\phi )\omega \\ +b_{22}\sin2(\alpha ,\phi )\omega +a_{32}\cos3(\alpha ,\phi )\omega +b_{32}\sin3(\alpha ,\phi )\omega \end{array}$$

(9) $$\begin{array}{r} M_{34}(\alpha ,\phi )=a_{03}+a_{13}\cos(\alpha ,\phi )\omega +b_{13}\sin(\alpha ,\phi )\omega +a_{23}\cos2(\alpha ,\phi )\omega \\ +b_{23}\sin2(\alpha ,\phi )\omega +a_{33}\cos3(\alpha ,\phi )\omega +b_{33}\sin3(\alpha ,\phi )\omega \end{array}$$

where the Fourier constants are as follows:

$$\begin{array}{c} a_{01},a_{11},b_{11},a_{21},b_{21},a_{31},b_{31},a_{02},a_{12},b_{12},a_{22},b_{22},a_{32},b_{32},a_{03},a_{13},b_{13},a_{23},\\ b_{23},a_{33},b_{33},a_{03},a_{13},b_{13},a_{23},b_{23},a_{33},b_{33},\omega \end{array}$$

### 2.3. The 3D WPT System Input Power in Terms of the Receiver Angles

By employing the attained mutual coupling angular functions of the system in the current load Equation (6), it is possible to establish a mathematical relationship that describes the power variation of the system in terms of the angular functions of the receiver trajectory. By substituting (7)–(9) in (6), the current load formula becomes:

(10) $$\begin{array}{l} i_{4}=\frac{jwI}{R_{4}+R_{L}+jX_{4}}\times \\ \left(M_{14}(\alpha ,\phi )\sin\beta \cos\theta +M_{24}(\alpha ,\phi )\sin\beta \sin\theta +M_{34}(\alpha ,\phi )\cos\beta \right) \end{array}$$

We define the power load formula as follows:

(11) $$P_{L}=\left|R_{L}\right|\times {\left|i_{4}\right|}^{2}$$

The amplitude of $i_{4}$ is defined by Equation (12)

(12) $$\begin{array}{l} \left|i_{4}\right|=\frac{wI}{\sqrt{{\left(R_{4}+R_{L}\right)}^{2}+X_{4}^{2}}}\times \\ \left|\left(M_{14}(\alpha ,\phi )\sin\beta \cos\theta +M_{24}(\alpha ,\phi )\sin\beta \sin\theta +M_{34}(\alpha ,\phi )\cos\beta \right)\right| \end{array}$$

The load power angular Equation (13) is obtained by substituting Equations (12) into (11).

(13) $$\begin{array}{l} P_{L}=\frac{w^{2}I^{2}}{{\left(R_{4}+R_{L}\right)}^{2}+X_{4}^{2}}\times \\ {\left(M_{14}(\alpha ,\phi )\sin\beta \cos\theta +M_{24}(\alpha ,\phi )\sin\beta \sin\theta +M_{34}(\alpha ,\phi )\cos\beta \right)}^{2} \end{array}$$

**Figure 7:**
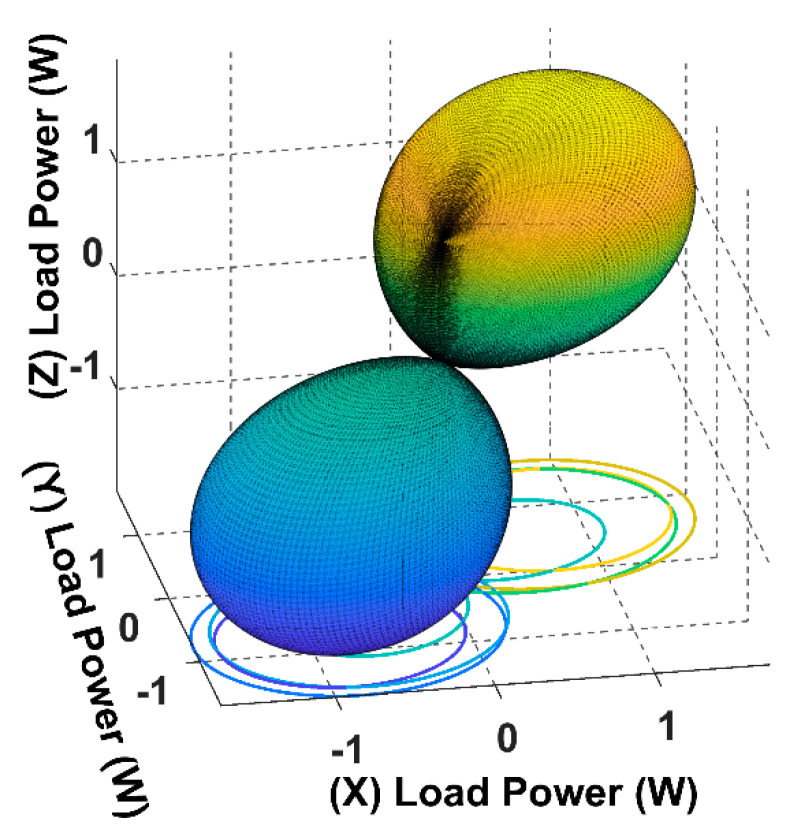
The power load spherical plot for the 3D WPT system when the receiver is placed at α = *ϕ* = 45°.

As it is showing in Figure 7, when the receiver is placed at α = *ϕ* = 45°and the magnetic field resultant is directed to the receiver position *θ* = *β* = 45°, the load power reaches its maximum for the 3D WPT.

Because of the receiver mobility, it is impractical to use the load power as feedback for the controlling process, therefore deriving the input power expression is a must.

The input power is defined as follows:

(14) $$P_{in}=P_{\mathrm{los}1}+P_{los2}+P_{\mathrm{los}3}+P_{los4}+P_{L}$$

where

(15) $$\begin{array}{l} P_{los1}=R_{1}\times i_{x}^{2}=R_{1}{\left(I\times \sin\beta \cos\theta \right)}^{2}\\ \;P_{los2}=R_{2}\times i_{y}^{2}=R_{2}{\left(I\times \sin\beta \sin\theta \right)}^{2}\\ \;P_{los3}=R_{3}\times I_{z}^{2}=R_{3}{\left(I\times \cos\beta \right)}^{2}\\ \;P_{los4}=R_{4}\times i_{4}^{2}\\ P_{in}=R_{1}{\left(I\times \sin\beta \cos\theta \right)}^{2}+R_{2}{\left(I\times \sin\beta \sin\theta \right)}^{2}\\ +R_{3}{\left(I\times \cos\beta \right)}^{2}+R_{4}\times {\left|i_{4}\right|}^{2}+R_{L}\times {\left|i_{4}\right|}^{2} \end{array}$$

By putting all the ohmic resistors of the coils equal to R and substituting (12) into (15) we have:

(16) $$\begin{array}{l} P_{in}=RI^{2}+(R_{4}+R_{L})\frac{w^{2}I^{2}}{{\left(R_{4}+R_{L}\right)}^{2}+X_{4}^{2}}\times \\ {\left(M_{14}(\alpha ,\phi )\sin\beta \cos\theta +M_{24}(\alpha ,\phi )\sin\beta \sin\theta +M_{34}(\alpha ,\phi )\cos\beta \right)}^{2} \end{array}$$

Equation (16) incorporates all the important parameters of the 3DWPT system. By introducing the angular parameters of the receiver trajectory (α, *ϕ*) and the magnetic field angles (*θ*, *β*) to the input power expression, we have established a comprehensive model able to simulate the system power with relation to the receiver dynamics for a particular trajectory.

The following input power curves are plotted for different receiver angular positions in Cartesian and spherical coordinates using the developed 3DWPT angular model as elaborated previously.

Using (16), we can simulate the input power curve when the receiver is fixed and the magnetic field resultant is rotating across the sending end, as shown in Figure 7.

**Figure 8:**
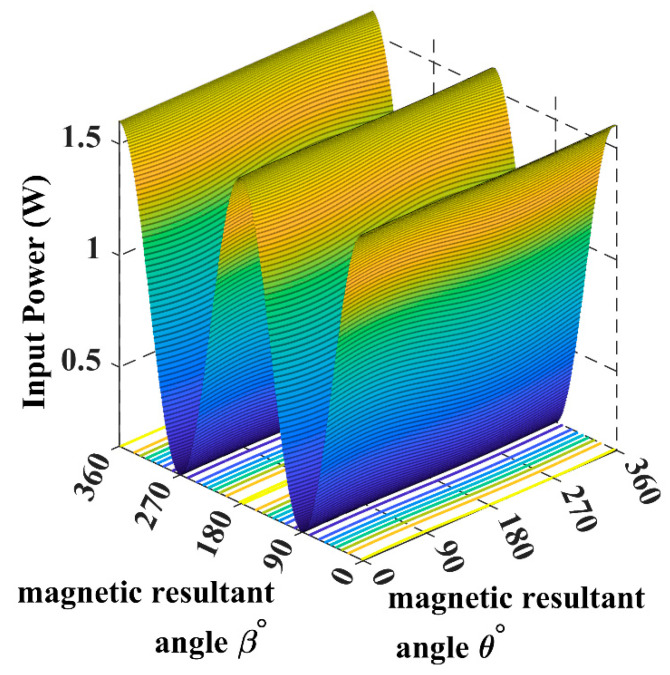
The input power plot for the 3D WPT system when the receiver is placed at α = *ϕ* = 0°.

**Figure 9:**
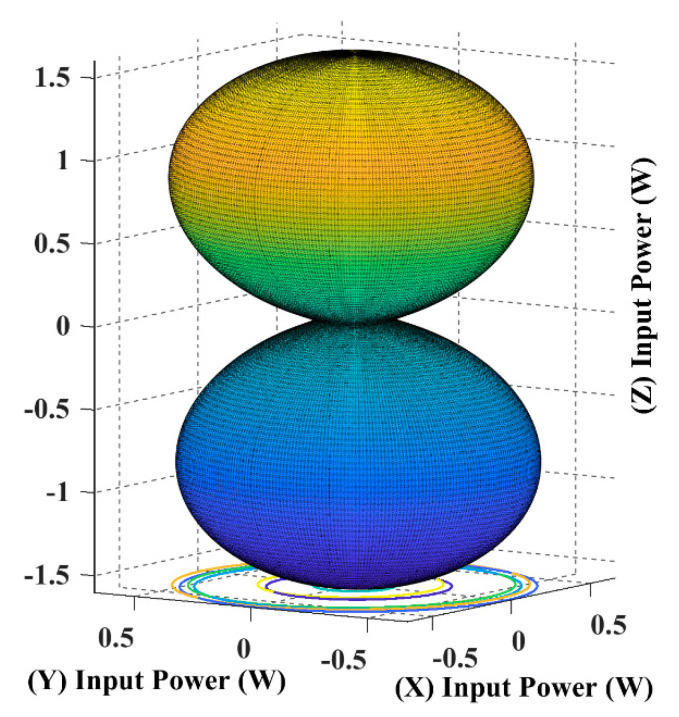
The input power spherical plot for the 3D WPT system when the receiver is placed at α = *ϕ* = 0°.

When the receiver is located on the top of the transmitting coils, facing the T_z_ coil, in this case, the maximum transferred input power is directed through the *Z*-axis, as shown in Figure 8 and Figure 9.

**Figure 10:**
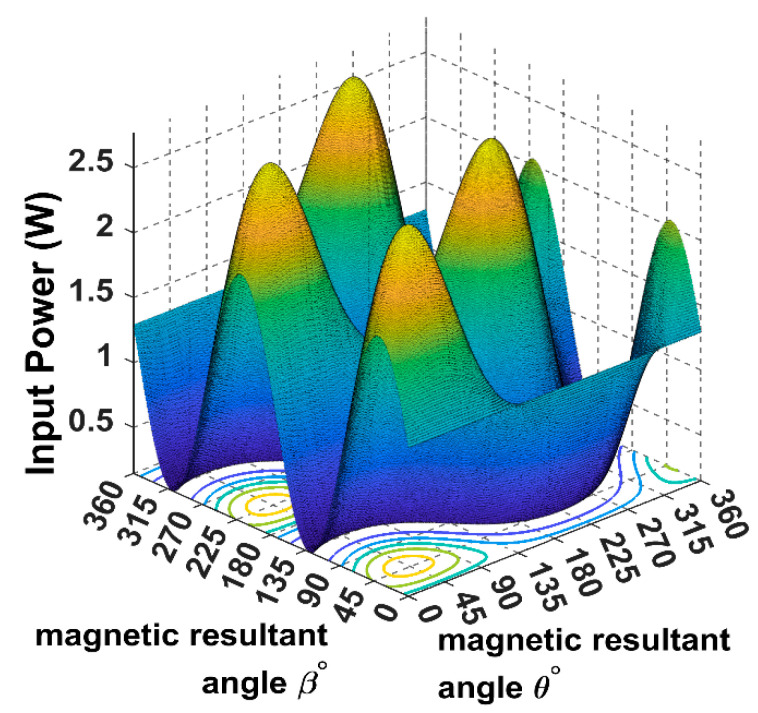
The input power plot for the 3D WPT system when the receiver is placed at α = *ϕ* = 45°.

From Figure 10, it is noticeable that when the receiver is placed at the 45° angular position, the power transfer reaches the maximum of 2.5 W towards the receiver direction. In this position, the receiver is coupled equally with the three transmitters, resulting in a significant rise in the input power.

**Figure 11:**
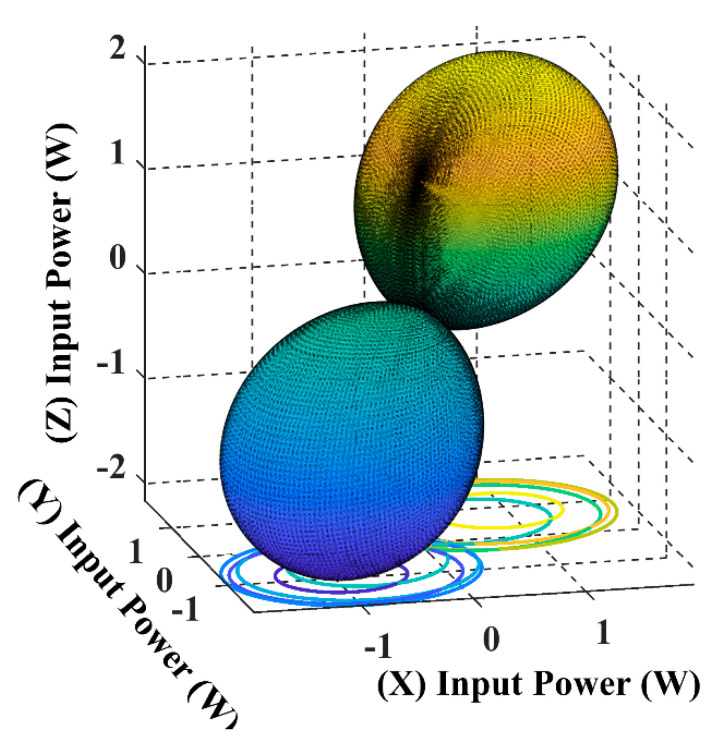
The input power spherical plot for the 3D WPT system when the receiver is placed at α = *ϕ* = 45°.

The spherical plot in Figure 11 shows the input power pattern when applying a rotating magnetic field on a fixed receiver position. It is noticeable that the symmetry in the input power peaks is due to the omnidirectional nature of the 3DWPT.

**Figure 12:**
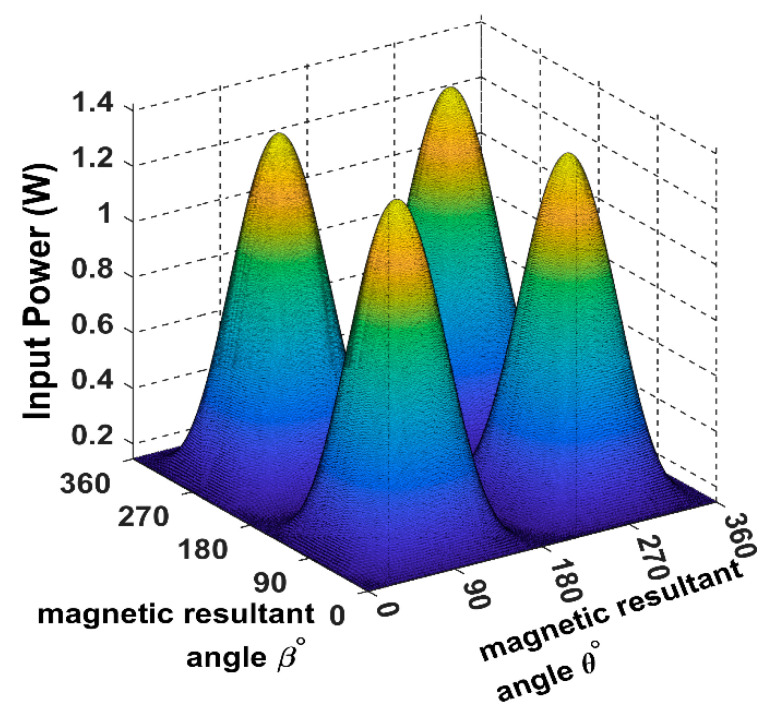
The input power plot for the 3D WPT system when the receiver is placed at α = *ϕ* = 90°.

**Figure 13:**
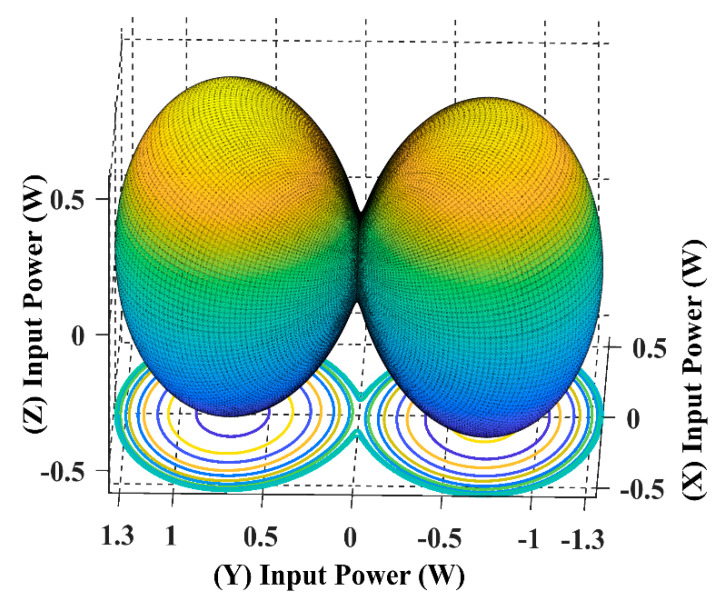
The input power spherical plot for the 3D WPT system when the receiver is placed at α = *ϕ* = 90°.

In Figure 12 and Figure 13, the receiver is fully coupled with Tx coil only, due to which the input power drops from 2.5 W to 1.4 W compared to the central position in Figure 11; the maximum input power flow is from the Tx coil to the receiver across the *Y*-axis.

**Figure 14:**
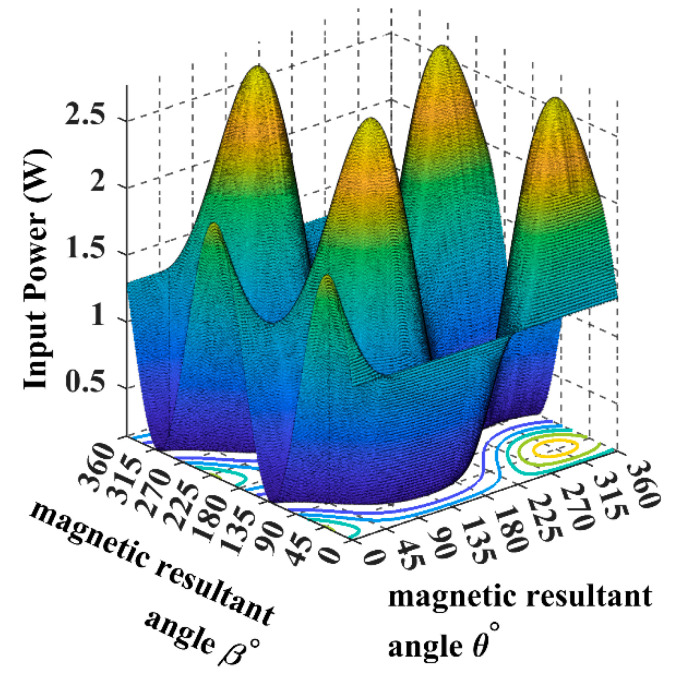
The input power plot for the 3D WPT system when the receiver is placed at α = *ϕ* = 135°.

By comparing Figure 14 with Figure 10 we can conclude that the 3DWPT system is genuine omnidirectional because the receiver will pick the same amount of power when it is placed in symmetric positions (α = *ϕ* = 45°, α = *ϕ* = 135°). 

**Figure 15:**
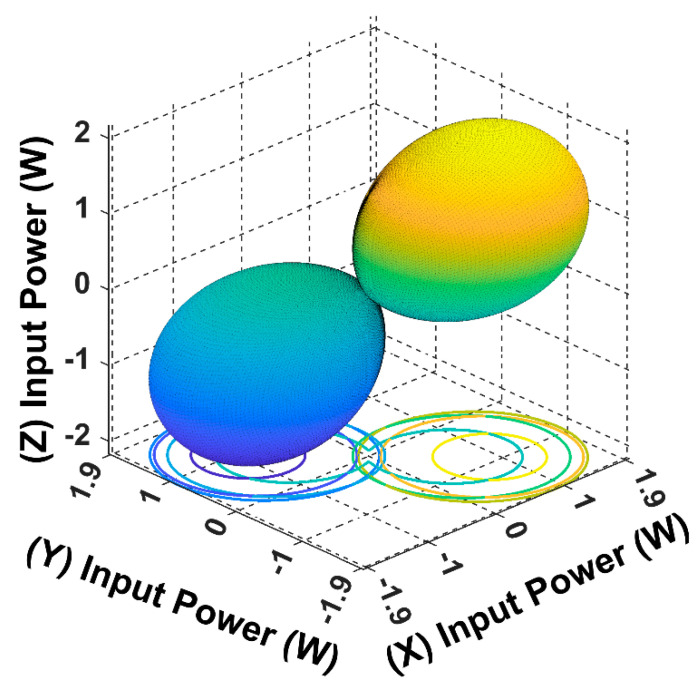
The input power spherical plot for the 3D WPT system when the receiver is placed at α = *ϕ* = 135°.

By observing Figure 11 and Figure 15, we can understand that both the receiver spherical positions when α = *ϕ* = 45°, α = *ϕ* = 135° produce the same input power of 2.5 W. However, the flow direction of the input power is focused on the 135° axis in the 3D plan.

The spherical plots of the input power in Figure 9, Figure 11, Figure 13 and Figure 15 are represented as a dumbbell shape with two maxima. Depending on the position of the receiver and the resultant magnetic angles *θ* and *β* in the spherical coordinates, the power transfer can be maximised through the directional control of the magnetic field resultant towards the exact position of the receiver. Table 4 in the benchmarking paper Ref. [16] shows the measured input power values when the receiver is coupled with one transmitting coil, which is similar to the cases when the receiver is positioned at α = *ϕ* = 0° and α = *ϕ* = 90° for this research.

By comparing Figure 8 and Figure 12 with Table 4, we can notice that the designed model in this paper is in close accordance with the measured value of the benchmarking paper. Since this paper proposes a non-collaborative control technique, all the feedback from the receiver is eliminated. By monitoring the input power measurement, we can sense the receiver position indirectly and use it to update the controller.

**Figure 16:**
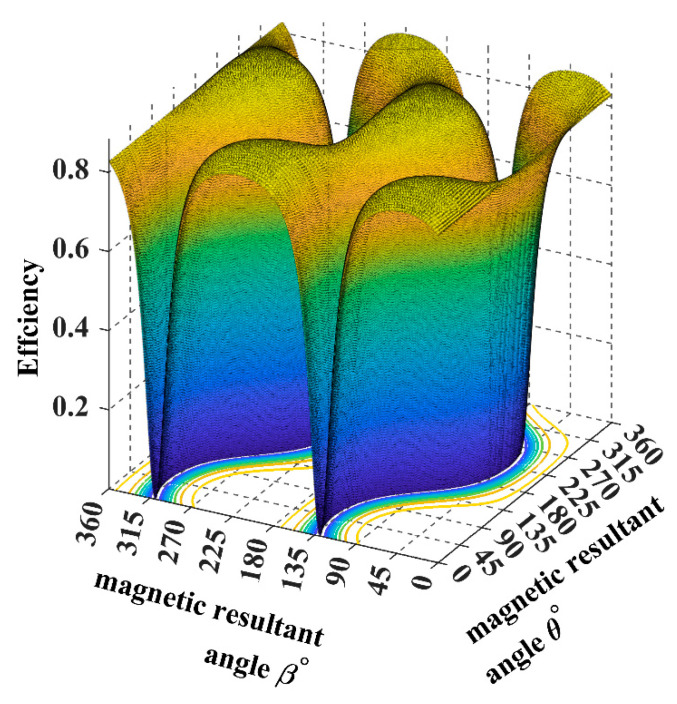
The system efficiency plot when the receiver is placed at 45°.

**Figure 17:**
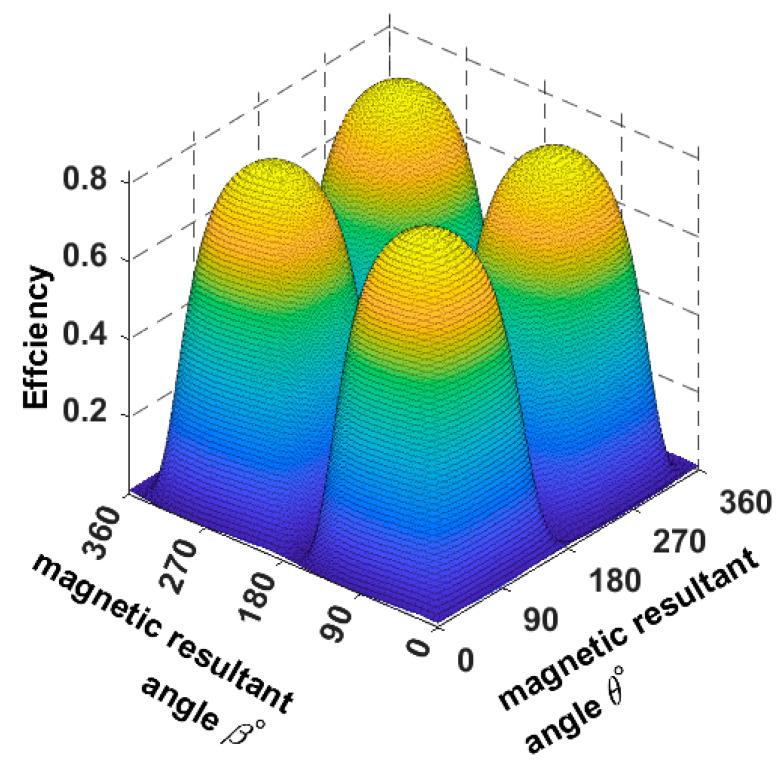
The system efficiency plot when the receiver is placed at 90°.

The plots in Figure 16 and Figure 17 show that the power transfer efficiency is up to 80% when the magnetic resultant points to the exact position of the receiver and 0% when the magnetic resultant is in a phase difference of 90° with the receiver. Thus, controlling the magnetic field resultant is a must to maintain power maximisation.

**Figure 18:**
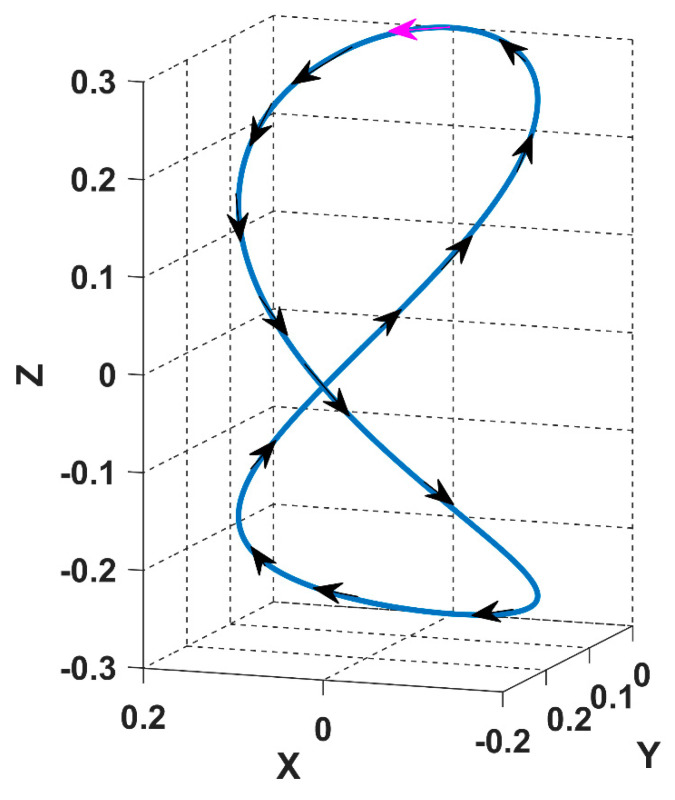
The 8-shaped receiver trajectory 𝝘 starts from the pink-coloured coil.

Figure 18 shows the studied trajectory when α and *ϕ* are varied with a 1°-degree step from 0° to 360°, starting and ending in the top position in pink colour.

**Figure 19:**
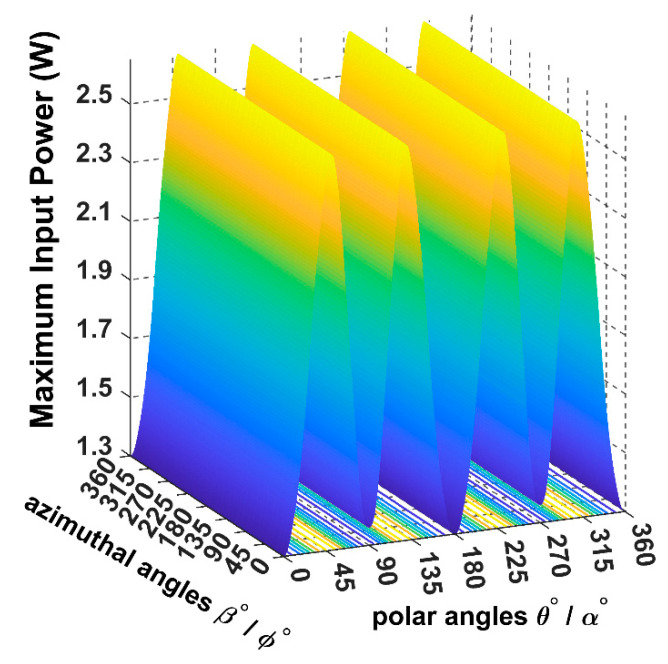
Maximum input power variation when the receiver angles and the magnetic angles are changed equally for the proposed trajectory.

**Figure 20:**
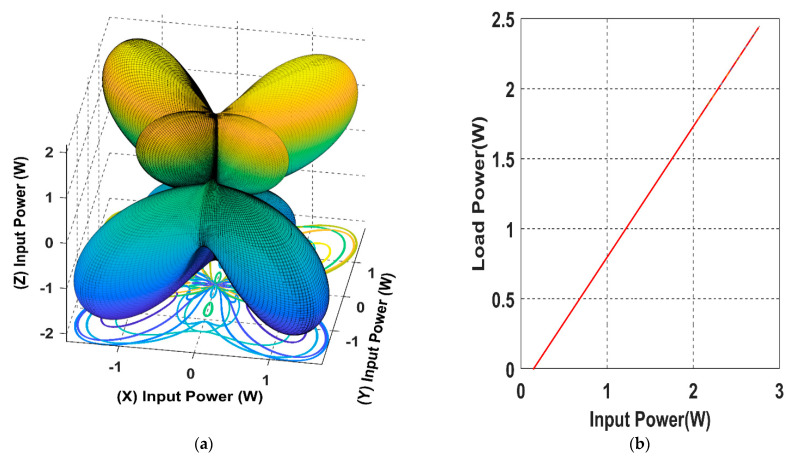
(**a**) The spherical plot of the calculated maximised input power when the receiver is moving according to the 8-shaped trajectory 𝝘. (**b**) The relationship between the input power and the load power for the 3D WPT system.

Figure 19 represents the maximum input power for the 3D WPT system when the receiver is moving according to the 8-shape continuous trajectory. The curve was simulated by rendering the functions of the magnetic resultant *θ*(t) and *β*(t), equal to the receiver angular continues trajectory α(t) and *ϕ*(t). As is depicted in Figure 20 by a blue and yellow colour, the input power fluctuates between two maxima of 1.3 W and 2.5 W, respectively.

The developed model in this work can generate the input power function at any given point from the chosen trajectory 𝝘. As shown in Figure 20a, the overall input power spherical plot for the 𝝘 trajectory is forming a butterfly shape. It is noticeable that the moving receiver can pick up power at any given point from 𝝘; however, it is necessary that the magnetic resultant angles are always equal to the receiver’s angular position regardless of its movement.

As we can notice from Figure 20b, the input power has a linear relationship with the load power, hence both the input power and the load power have the same variation behaviour with a different amplitude.

From this section, we conclude that if we can design a control action that can track magnetically the receiver position across the trajectory 𝝘, it is possible to ensure an autonomous maximised power transfer for a moving load in a 3D trajectory without feedback.

## 3. The Extremum Seeking Control Implementation for the 3D WPT System

### 3.1. Extremum Seeking Scheme for the Multi-Parameter System

According to (13), maximising the load power through the input power is possible. The only available feedback from the transmitter is the input power to maximise the power transfer efficiency. Thus, taking it as the objective function f(γ) for the extremum seeking controller (ESC) as follows:

By putting $f\left(\gamma \right)=P_{in}$, we have:

(17) $$\begin{array}{l} f\left(\gamma \right)=RI^{2}+(R_{4}+R_{L})\frac{w^{2}I^{2}}{{\left(R_{4}+R_{L}\right)}^{2}+X_{4}^{2}}\times \\ {\left(M_{14}(\alpha ,\phi )\sin\beta \cos\theta +M_{24}(\alpha ,\phi )\sin\beta \sin\theta +M_{34}(\alpha ,\phi )\cos\beta \right)}^{2} \end{array}$$

The continuously differentiable function can be approximated locally by Equation (18) as detailed in [20]:

(18) $$f\left(\gamma \right)=f^{*}(t)+{\left(\gamma -\gamma^{*}(t)\right)}^{T}P(\gamma -\gamma^{*}(t))$$

where f is a $C^{2}$ function, γ is the input and, $\gamma^{*}$ is the optimal value that renders the output equal to the extremum $f^{*}$. The objective is to minimise the difference between the unknown optimal input value and the current value so that γ it converges to $\gamma^{*}$ which makes $f\left(\gamma \right)=f^{*}$. $P_{l\times l}=P^{T}<0$ is a gain matrix, $\gamma ={\left[\gamma_{1}\ldots .\gamma_{l}\right]}^{T}$ and l indicates the number of the input parameters to the plant, in our case l=2 (the rotating magnetic field angles *θ*, *β*), $\gamma^{*}={\left[\gamma_{1}^{*}\ldots \ldots .\gamma_{l}^{*}\right]}^{T}={\left[\theta^{*},\beta^{*}\right]}^{T}$.

**Figure 21:**
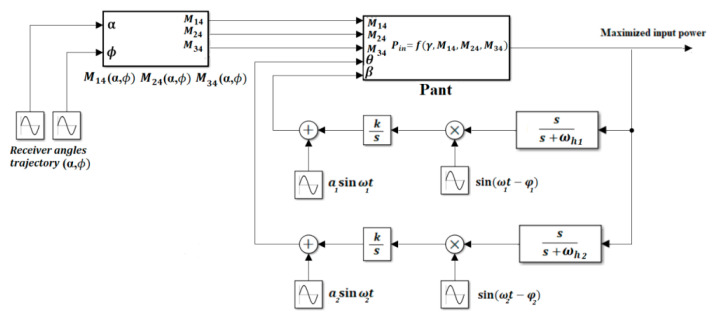
Extremum seeking scheme for 3D omnidirectional WPT power maximisation.

In the controller design for the 3DWPT system, we are using a multiparameter extremum seeking control with two inputs (*θ*, *β*) and one output, the input power *P*_in_. In order to simulate the receiver dynamics in the 3DWPT plant, the functions of the mutual coupling *M*_14_(α, *ϕ*), *M*_24_(α, *ϕ*), and *M*_34_(α, *ϕ*) are set to be varied during the tracking process of the controller. It is possible to simulate various movement patterns and velocities according to the defined trajectory in Figure 19 simply by selecting the desired signals of the receiver position angles α and *ϕ* in the spherical coordinates. Figure 21 illustrates the proposed scheme for the system simulation and control implementation.

The controller starts to search for the optimum input $\gamma^{*}$ values that maximise the objective function regardless of the mobility behaviour of the receiver. Since the power transfer is omnidirectional, the magnetic field angles (*θ*, *β*) control process is run only in the first half of the sphere of the spherical coordinates. Consequently, it will cover the other remaining half. The simulation results obtained by running a Simulink-based design scheme are shown in Figure 21, which accurately tracks the maximum input power with a small-time response for both steady-state and continuous rapid movement of the receiver.

### 3.2. The Closed-Loop Response When Using ESC for a Continuous Trajectory and Constant Velocity

**Figure 22:**
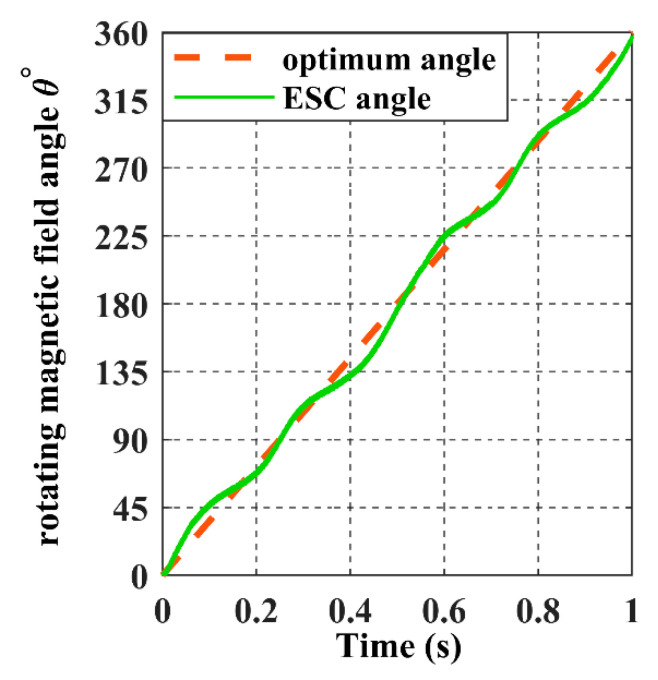
The rotating magnetic field angle *θ* in the closed-loop when the receiver moves according to the 8-shaped trajectory 𝝘.

**Figure 23:**
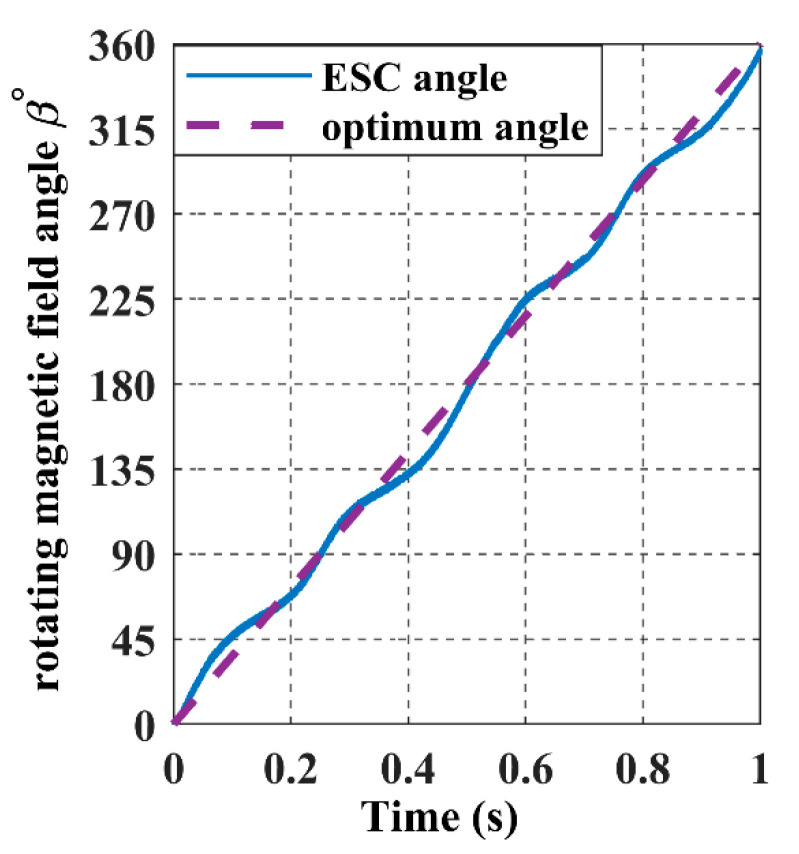
The rotating magnetic field angle *β* in the closed-loop when the receiver moves according to the 8-shaped trajectory 𝝘.

For the receiver to achieve the proposed trajectory in Figure 18, α and *ϕ* must be a ramp signal with a constant slope from 0° to 360°, as represented with dashed lines in Figure 22 and Figure 23.

**Figure 24:**
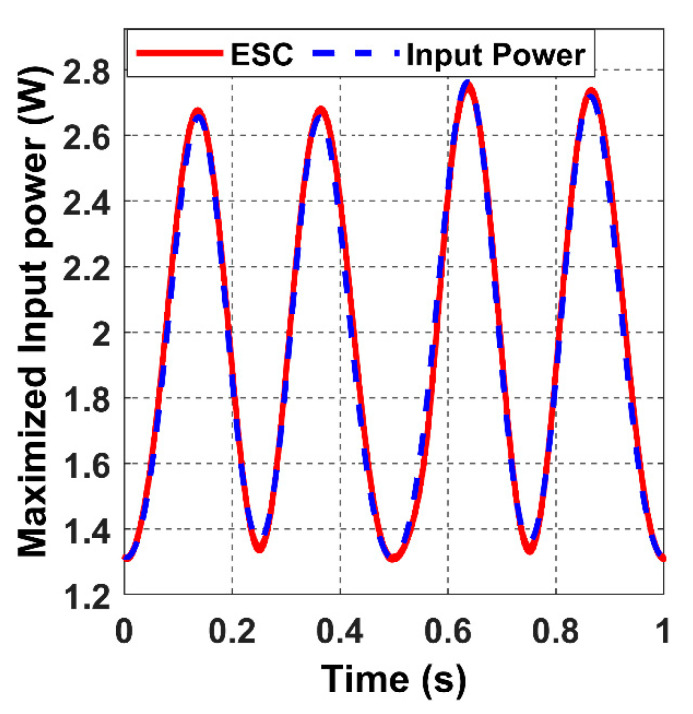
The system’s closed-loop output when the receiver moves in the 8-shaped trajectory 𝝘.

The ESC controller tracks the values of the receiver angles accurately by regulating the magnetic felid angles *θ* and *β* to be equal to α and *ϕ*, as it is shown in the above graph, which results in the maximisation of the input power, as shown in Figure 24.

Figure 24 shows that the input power fluctuates with a constant frequency due to the constant velocity of the receiver.

### 3.3. The Closed-Loop Response When Using ESC for a Continuous Trajectory with Intermittent Movement

**Figure 25:**
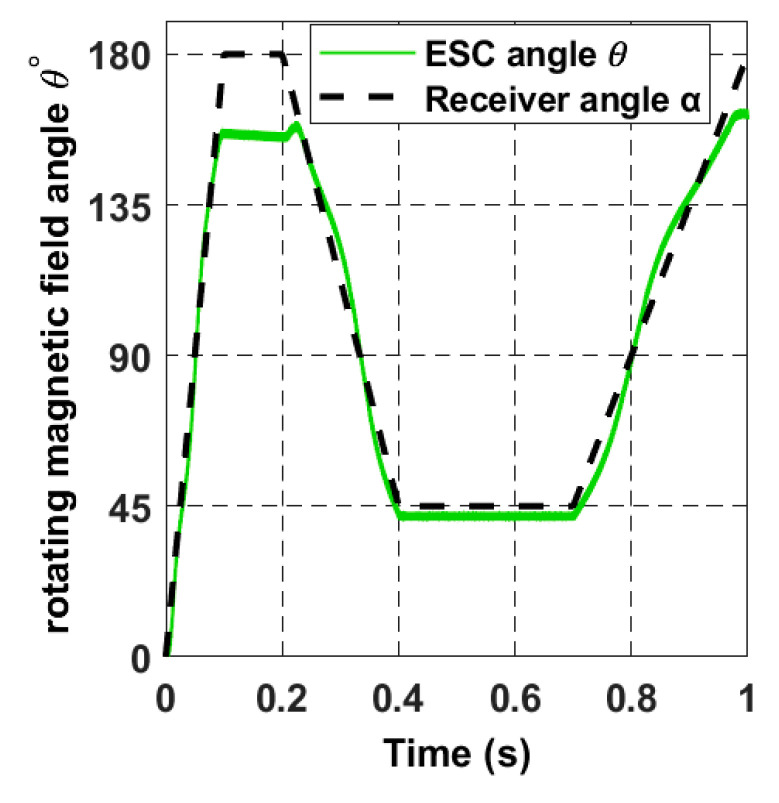
The rotating magnetic field angle *θ* in the closed-loop when the receiver moves intermittently.

**Figure 26:**
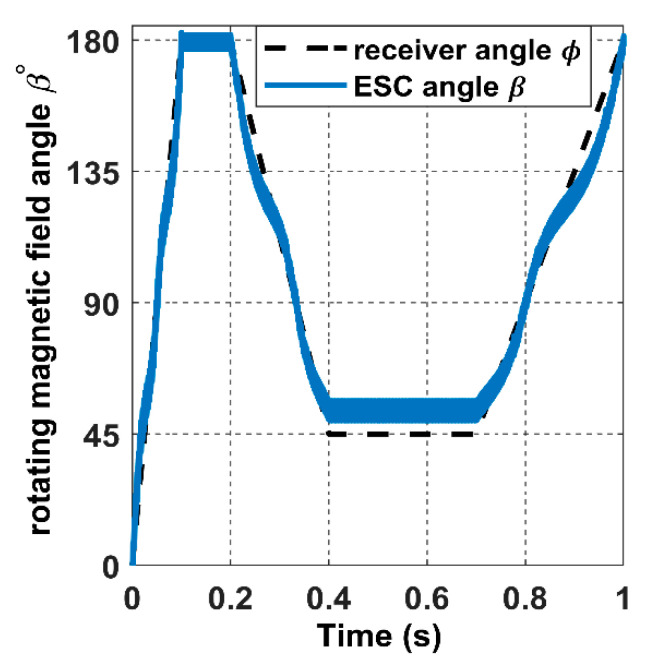
The rotating magnetic field angle *β* in the closed-loop when the receiver moves intermittently.

In this section, we simulate an intermittent motion for the receiver dynamics. We use the following angular signal for the receiver trajectory block as input for α and *ϕ*; the signal consists of a movement at a fixed angular speed of 31.4 rad/s from 0 s to 0.1 s. After that, the receiver takes a static position from 0.1 s to 0.2 s. The movement sequence is repeated, as shown in Figure 25 and Figure 26. The black dashed line represents the trajectory, as mentioned earlier, of the receiver in the spherical coordinates, while the green and blue signals represent the searched magnetic angles *θ* and *β*, respectively.

**Figure 27:**
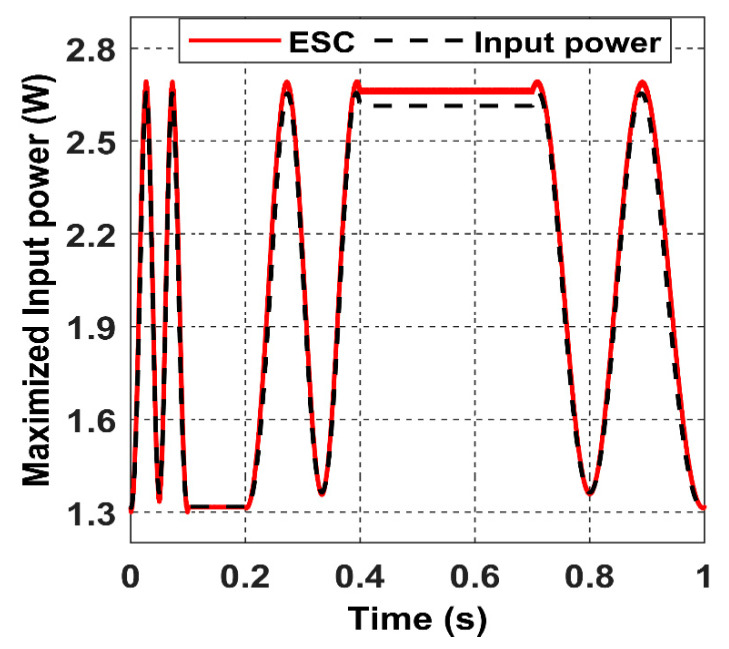
The maximised input power response in the closed-loop versus the calculated input power when the receiver moves intermittently.

When applying an interment movement on the receiver, we can notice that the multi-parameter extremum seeking control technique can preserve an accurate tracking of the maximum power under instantaneous movement transitions, as shown in Figure 27.

The receiver accelerated movement is reflected in the input power graph, where the frequency of the input power signal is increased in the time interval: 0 s to 0.1 s.

**Figure 28:**
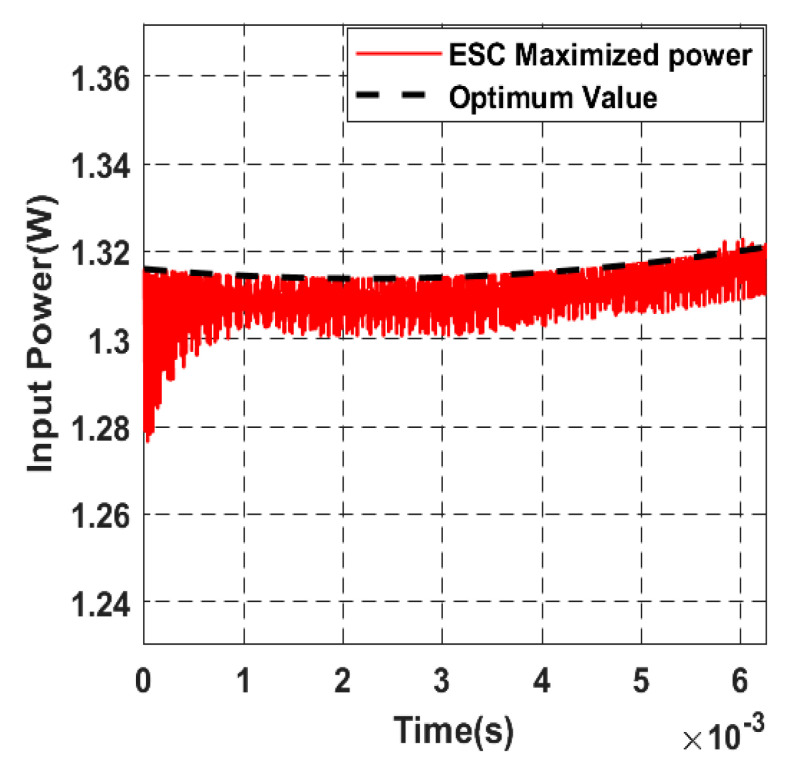
Zoomed view shows how the closed-loop input power converges to the maximum.

Figure 28 shows the tracking process of the maximum input power, as we notice the controller can reach the optimum in a small-time response of 1ms with a small steady-state error.

**Figure 29:**
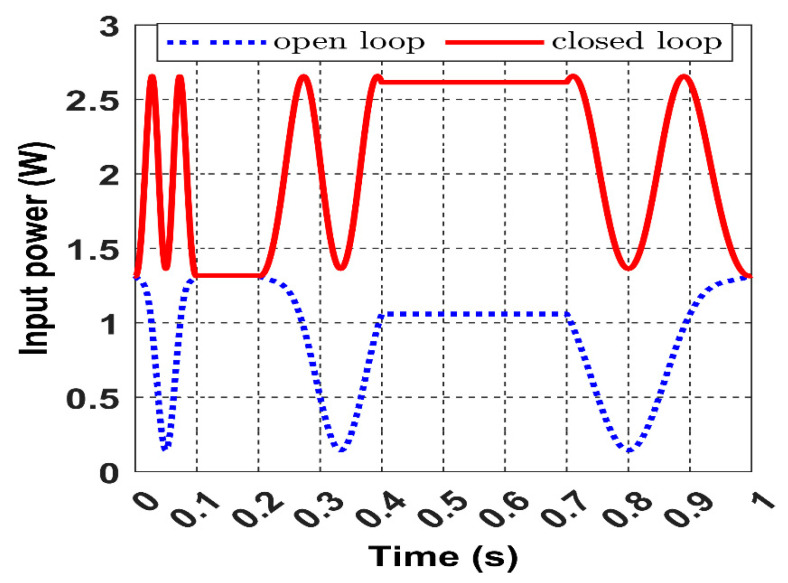
The input power comparison between the closed-loop and the open-loop.

In Figure 29, the difference between the open- and closed-loop input power is quite remarkable; the controller will continuously track the maximum available power for each receiver position.

### 3.4. The Closed-Loop Response When Using ESC for a Continuous Trajectory with an Accelerated Movement

**Figure 30:**
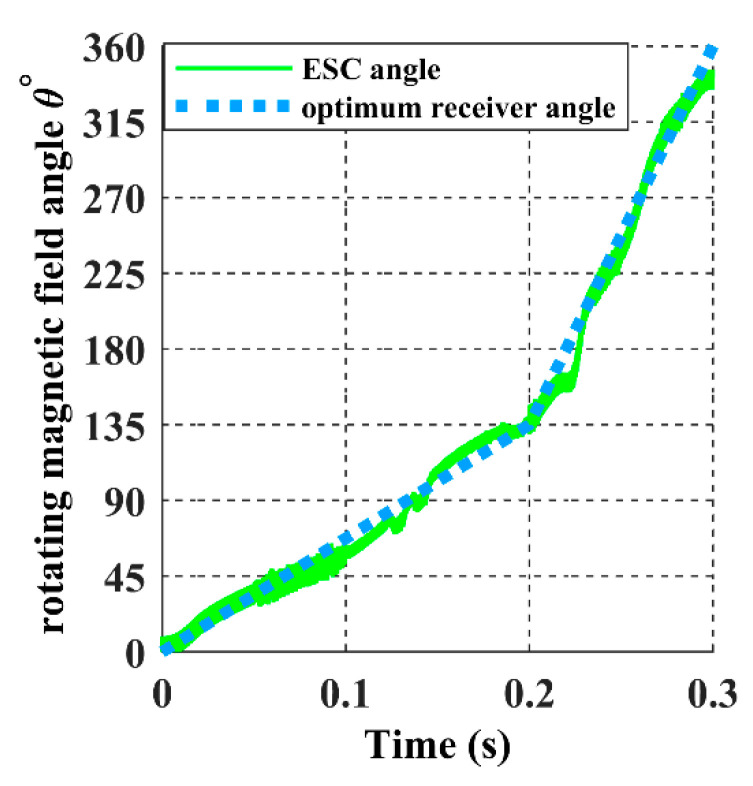
The rotating magnetic field angle θ in the closed-loop when the receiver is on accelerated movement.

**Figure 31:**
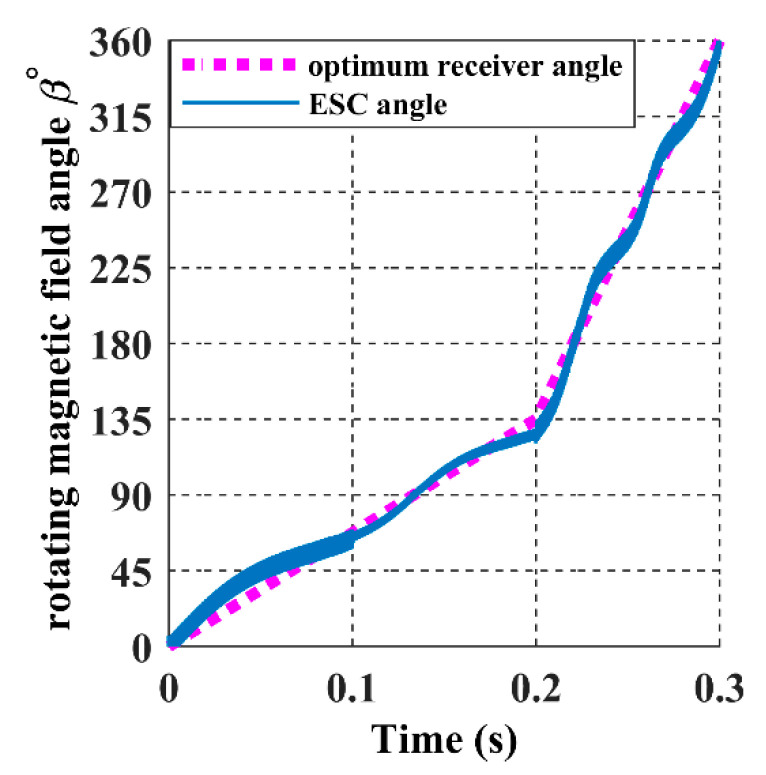
The rotating magnetic field angle β in the closed-loop when the receiver is on accelerated movement.

This section examines the effect of the receiver’s accelerated movement on the 3DWPT system in the closed loop. By increasing the ramp slope of the receiver angular input signals α and *ϕ*, one can increase the velocity and make it variable. From 0 (s) to 0.2 (s), the receiver rotates from 0° to 135° with a velocity of 675 deg/s. After that, the velocity is increased to 2250 deg/s, as shown in dashed lines in Figure 30 and Figure 31.

**Figure 32:**
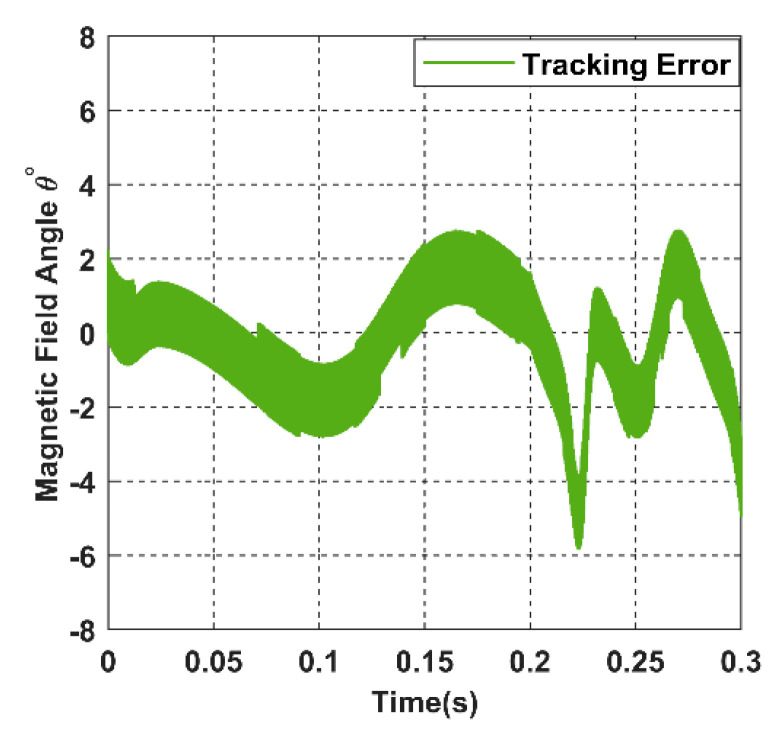
The tracking error for the magnetic angle *θ*.

The green signal in Figure 30 represents the optimum value of the magnetic field polar angle *θ* searched by the controller, which maximises the input power. As we can see, the value of *θ* always oscillates around the exact receiver trajectory α, which ensures accurate magnetic tracking for the moving target with small angular error, as shown in Figure 32.

**Figure 33:**
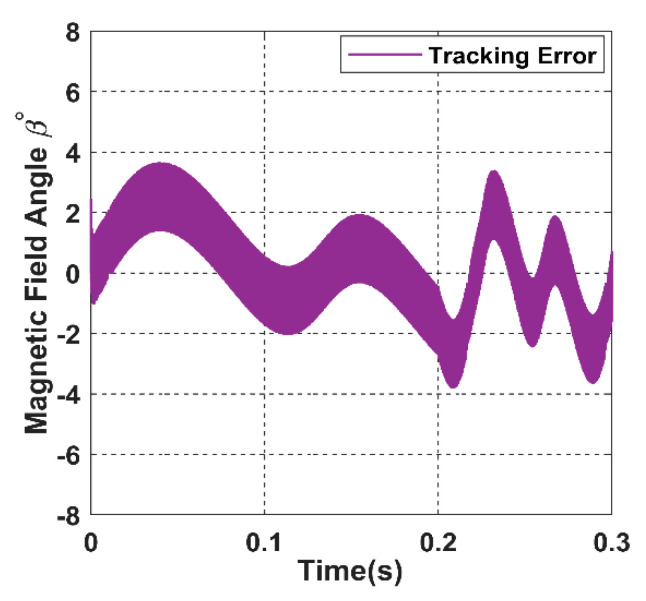
The tracking error for the magnetic angle *β*.

Similarly, in the closed loop, the magnetic field azimuthal angle *β* tracks the angular receiver position *ϕ* precisely, as shown in blue colour from Figure 31. Correspondingly, the tracking angle error of *β* fluctuates between −4° and 4°in Figure 33, which is considered a minor value due to the calculation error present in the model.

**Figure 34:**
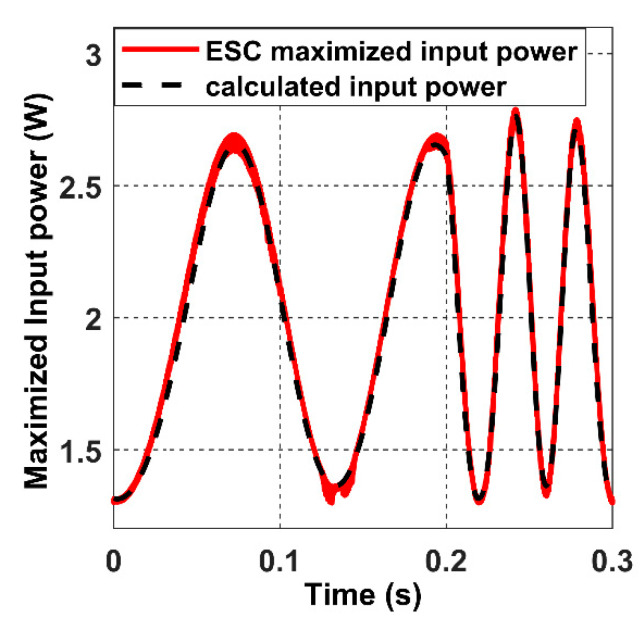
The maximised input power closed-loop response under an accelerated receiver movement.

The calculated input power in the black dashed line in Figure 34 shows how the input power range alternates from partially coupled to a fully coupled receiver under an accelerated movement at high velocity (simulation time 0.3 s). When the closed-loop ES control action is applied, the red curve represents the maximised input power searched by the controller. When the acceleration occurs at 0.3 s, the frequency of the input power signal increase due to the rise in the receiver velocity.

From Figure 31, it is observable that the receiver will pick up the maximum power regardless of its position or speed under the closed-loop control. Hence the maximisation of the power transfer for the 3D orthogonal coils system is achieved for rapidly accelerated mobility. The developed controller is able to attain a maximum input power output for the 3D WPT system in a very small-time response and almost insignificant static error.

**Figure 35:**
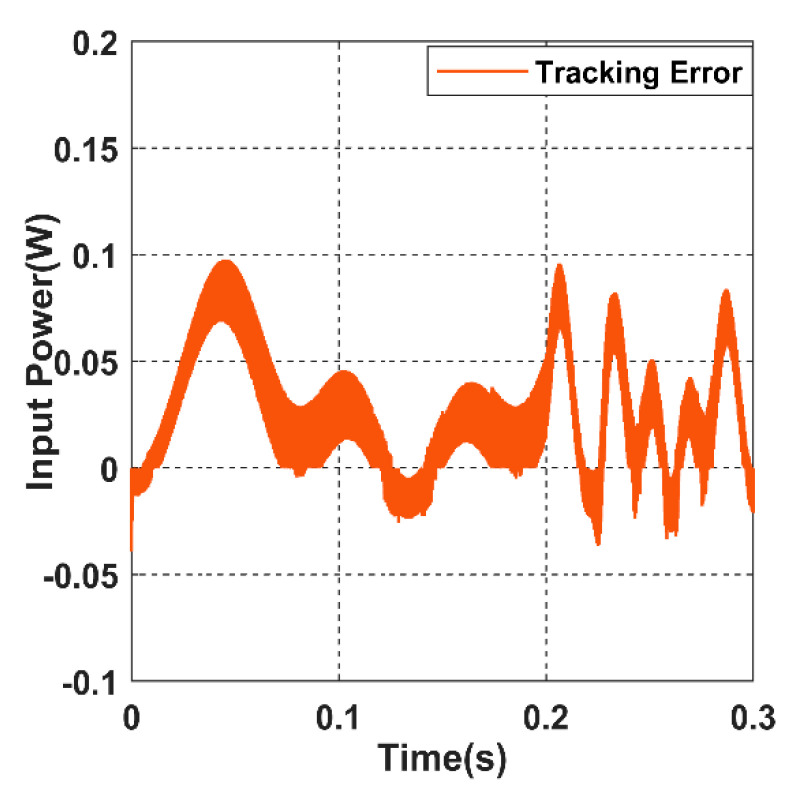
The tracking error of the input power.

Figure 35 shows the values of the tracking error, which is less than 0.1 w. It is noticeable that when the receiver velocity increased in the last region of the simulation time, the frequency of the error picks rises as well.

Table 5 summarises the limitations as well as the contribution of each developed control method for the 3dWPT system. Both references [15,16] do not take into consideration the mobility of the receiver, neither the continuous trajectory or the velocity.

**Figure 36:**
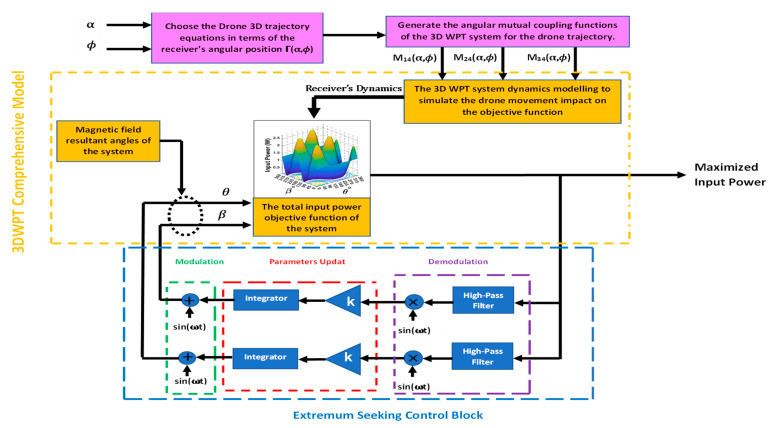
A general flowchart that summarises the 3DWPT model and the control method.

In Figure 36, we have encapsulated the modelling steps of the receiver trajectory along with the 3DWPT transmitter. First, we define a trajectory for the drone using angular positions; after that, the dynamic model will generate the angular mutual coupling functions for the used trajectory. After that, mutual coupling drone’s dynamics is implemented in the objective function of the input power (electric model of the 3DWPT).

Furthermore, the ES control loop is also presented. The value of the gradient change for the plant is probed by the sinusoidal perturbation signal that is fed into the system’s measured output.

K is the learning rate which determines the convergence speed to the maximum, whereas the high pass filter is used to reduce the static error as well as ensure the stability of the closed-loop response.

## 4. Conclusions

This paper presented a design control and modelling for a three-dimensional wireless power transfer system with dynamic charging for mobile loads. Modelling an extensive WPT plant system incorporating the receiver mobility in a 3D trajectory will contribute to a relabel control design for the magnetic tracking concept. Based on the obtained model, an extremum seeking controller is integrated to optimise the input power function under high-speed receiver motion. The controller was tested at a variable velocity receiver trajectory, including the accelerated and intermittent movement of the load. In the closed loop, the input power converged with the calculated maximum of 2.6 W in less than 1ms for the studied 8-shaped trajectory. When the receiver rotates, the maximum power is guaranteed for an angular velocity of 2250 deg/s. In theory, the control method is suitable for intelligent applications such as drones and smart sensors. The ESC is effective in response time and steady-state error, providing a maximum power transfer regardless of the receiver’s position, trajectory, or speed.

## Figures and Tables

**Table 1:** 3D WPT System coils parameters.

Coils	Turns	The Current Resultant *I* (A)	Frequency (kHz)	Coils Resistance R (Ω)	L (µH)	C (ηF)	Coils Radius(m)	Load Resistance RL (Ω)
T_x_	11	0.45	550.77	0.72	82.03	1	0.3	
T_y_	11	0.45	550.77	0.72	82.03	1	0.3	
T_z_	11	0.45	550.77	0.72	82.03	1	0.3	
Receiver	11		550.77	0.72	82.03	1	0.3	10

**Table 2:** Mutual coupling values Ref. [16].

*θ* (deg)	*M_x_-L*_2_	*M_y_-L*_2_	*M_z_-L*_2_
45	2.372 µH	2.361 μH	~0
90	~0	2.579 μH	~0
180	−2.607 μH	~0	~0

**Table 3:** Mutual coupling values of this work.

*θ*/*ϕ* (deg)	*M*_14_	*M*_24_	*M*_34_
45	2.009 μH	2.009 μH	2.009 μH
90	2.319 μH	~0	~0
180	~0	~0	−2.27 μH

**Table 4:** Measured values of input power Ref. [16].

P_*in*_				P_*out*_
P_*inx*_ (W)	P_*iny*_ (W)	P_*inz*_ (W)	P_*intotal*_ (W)	
0.29	1.21	0.04	1.54	1.07

**Table 5:** Comparative methods table.

	Ref. [16]	Ref. [15]	This Work
Method	Scanning method	Mathematical method	Multiparameter Extremum Seeking Control algorithm
System	3D-WPT	3D-WPT	3D-WPT
Control Method	weighted time-sharing	Mathematical	Continuous Optimisation
Transmitting Coil structure	Orthogonal circular coils	Orthogonal circular coils	Orthogonal circular coils
Algorithm implementation	Complex, poor expensive	Complex, poor, expensive	Simple computation no control unit is needed no discretisation or signal processing is needed.
Receiver dynamics (Continuous trajectory)	Not covered	Not covered	Continues rotational trajectory
Receiver dynamics (velocity acceleration)	Not covered	Not covered	Covered
